# Evaluating determinants of receipt of molecular imaging in biochemical recurrent prostate cancer

**DOI:** 10.1002/cam4.3555

**Published:** 2020-11-28

**Authors:** Hala T. Borno, Tracy Kuo Lin, Anobel Y. Odisho, Arpita Desai, Vadim Koshkin, Kalin Werner, Nichole Legaspi, Matthew Bucknor, Alexander Bell, Sylvia Zhang, Thomas A. Hope

**Affiliations:** ^1^ Department of Medicine Division of Hematology/Oncology University of California San Francisco San Francisco CA USA; ^2^ Department of Social and Behavioral Sciences Institute for Health & Aging University of California San Francisco San Francisco CA USA; ^3^ Department of Urology University of California San Francisco San Francisco CA USA; ^4^ Center for Digital Health Innovation University of California San Francisco CA USA; ^5^ Division of Emergency Medicine University of Cape Town Cape Town South Africa; ^6^ Department of Radiology and Biomedical Imaging University of California San Francisco San Francisco CA USA; ^7^ School of Medicine University of California San Francisco San Francisco CA USA

**Keywords:** biochemical recurrence, disparities, medical oncology, molecular imaging, prostate cancer

## Abstract

**Background:**

Molecular imaging with novel radiotracers is changing the treatment landscape in prostate cancer (PCa). Currently, standard of care includes either conventional and molecular imaging at time of biochemical recurrence (BCR). This study evaluated the determinants of and cost associated with utilization of molecular imaging for BCR PCa.

**Methods:**

This is a retrospective observational cohort study among men with BCR PCa from June 2018 to May 2019. Multivariate logistic regression models were employed to analyze the primary outcome: receipt of molecular imaging (e.g. Fluciclovine PET and Prostate Specific Membrane Antigen PET) as part of diagnostic work‐up for BCR PCa. Multivariate linear regression models were used to analyze the secondary outcome: overall healthcare cost within a 1‐year time frame.

**Results:**

The study sample included 234 patients; 79.1% White, 2.1% Black, 8.5% Asian/Pacific Islander, and 10.3% Other. The majority were 55 years or older (97.9%) and publicly insured (74.8%). Analysis indicated a one‐unit reduction in PSA is associated with 1.3 times higher likelihood of receiving molecular imaging (*p* < 0.01). Analysis found that privately insured patients were associated with approximately $500,000 more in hospital reimbursement (*p* < 0.01) as compared to the publicly insured. Additionally, a one‐unit increase in PSA is associated with $6254 increase in hospital reimbursement or an increase in total payments by 2.1% (*p* < 0.05).

**Conclusions:**

Higher PSA was associated with lower likelihood for molecular imaging and higher cost in a one‐year time frame. Higher cost was also associated with private insurance, but there was no clear relationship between insurance type and imaging type.

## Graphical abstract

This study evaluated the determinants of and cost associated with utilization of molecular imaging for biochemically recurrent prostate cancer. Higher prostate‐specific antigen level was associated with lower likelihood for molecular imaging and higher cost in a 1‐year timeframe.

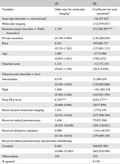

## 1 BACKGROUND

Prostate cancer (PCa) is the second leading cause of cancer‐related deaths among men in the United States.^1^ Among men who undergo treatment for localized PCa, approximately 20% will develop subsequent biochemical recurrence (BCR) or a rising serum prostate‐specific antigen (PSA).^2^, ^3^ In the era of precision medicine, emerging use of novel radiotracers such as 68‐Gallium Prostate Specific Membrane Antigen (PSMA) and 18‐Fluciclovine positron emission tomography (PET) has led to an enhanced ability to characterize the extent of disease in men with low volume BCR PCa.^4^ Fluciclovine PET was approved by the United States Food and Drug Administration in May 2016,^5^ and by extension became covered for the Medicare insured by 2017.^6^ Wider availability of such molecular imaging modalities has recently been recognized to create a “stage shift” in PCa,^7^ specifically identifying metastatic disease in cases that would be considered non‐metastatic based on conventional imaging (e.g. computed tomography scans, nuclear medicine bone scan).

As a result, currently, molecular imaging is an important component of PCa management as it gives greater detail regarding extent of disease. Characterizing the extent of disease in the setting of BCR may lead patients to more intensified hormonal therapy with androgen signaling inhibitors combined with androgen deprivation therapy (ADT) and consideration of metastases‐directed therapy or targeted radiation to oligo‐metastases.^8^ Moreover, clinical trials are increasingly incorporating molecular imaging modalities into screening and surveillance protocols. However, whether these technologies are utilized equitably by race/ethnicity and other social factors is poorly understood.

This study sought to identify factors that best predict receipt of molecular imaging in the context of BCR PCa leveraging a real‐world data set.

## 2 METHODS

### 2.1 Data collection

This study is a retrospective observational cohort study within the University of California, San Francisco (UCSF). Data were obtained among all men with BCR PCa seen at UCSF from June 2018 to May 2019, regardless of histologic subtype (*N* = 234). Sociodemographic data were collected from medical records. Claims data were collected to estimate healthcare cost. This study received Institutional Review Board approval at UCSF, and informed consent was waived.

### 2.2 Outcome variables

The primary outcome of this analysis was receipt of molecular imaging (Fluciclovine PET and PSMA scan) as part of diagnostic work‐up for BCR PCa. PSMA scans were available as part of an investigational imaging study at UCSF; the inclusion criteria aligned with standard of care, therefore PSMA scans were included in this analysis to provide insight on utilization patterns. Diagnostic scan type was derived from chart review data and diagnostic scans and recurrence scans were grouped into separate categories. Instances where no diagnostic scans were completed were removed from the analysis.

The secondary outcome was healthcare cost, which captures the overall outpatient healthcare cost, measured by hospital reimbursement, over a 1‐year time frame. The costs were estimated using itemized payment amount from UCSF claims data for outpatient care. Only claims that were paid were included in the analysis as nonzeroed out data were not available for analysis and therefore excluded. Inpatient costs were excluded because inpatient healthcare services are billed by capitation and are often not itemized, thus preventing the evaluation of cost. We were able to obtain all claims for patients in the study sample and thus assumed that there were no patients in the study sample who were unable to pay for the healthcare services received. The claims data collected for the cohort included insurance coverage, total charges and total payments. Patients without insurers were identified as Self‐Paying the claims data and therefore their charges were considered out‐of‐pocket (OOP) costs. Subsequent prescriptions received were collected through billing claims and verified through medical record review.

### 2.3 Clinical characteristics

All patients had prior definitive prostate‐directed therapy with either radical prostatectomy (RP) (32.1%, *n* = 75), prostate radiotherapy (26.9%, *n* = 63), or RP followed by radiation therapy (41.0%, *n* = 96); patients who only received ADT monotherapy (*n* = 11) were excluded from the analysis. We measured comorbidity burden using Charlson comorbidity score derived from the electronic medical record data.^9^ Overall Gleason score was grouped into less than or equal to 6 (low), Gleason score 7 (medium), or Gleason score 8–10 (high).

Patients’ prior PSA levels were extracted from chart review. If a patient has no PSA level listed immediately before imaging, the PSA level reported prior and closest to the imaging date were incorporated for analysis. If a patient has no PSA level report prior to imaging, the reported baseline PSA level was used as prior PSA level.

Receipt of ADT during definitive therapy or prior to BCR was collected from the medical record. Definitive treatment was defined as the last date of the following variables: RP date, definitive radiation therapy date, or the end date of the last radiation following RP.

Last radiation therapy was calculated using the latest date of any radiation therapy recorded for the patient. Some patients later experienced a recurrence and received further treatment of either ADT, or radiation therapy (62.4%, *n* = 146). ADT was more common (57.3%, *n* = 134) than radiation therapy after imaging (23.1%, *n* = 54). Further treatment is out of the scope of this study and therefore management of patients following scans was not included in the analyses.

### 2.4 Statistical analysis

The utilization of molecular imaging among men with BCR PCa was examined. Differences in the characteristics between men who received molecular imaging and conventional imaging (defined as computed tomography, magnetic resonance imaging, or technetium‐99 nuclear medicine bone scans) was compared using chi square tests. The association of race/ethnicity with receipt of molecular imaging was modeled using a multivariate logistic regression. Men who received both molecular and conventional imaging were included with those who received molecular imaging. Covariates considered for inclusion in the multivariable model were race/ethnicity, age at time of analysis, Gleason score, PSA value at time of recurrence, primary healthcare payer, Charlson comorbidity score, and receipt of prior molecular imaging. A purposeful selection strategy, which involved extensive discussions with oncologists on the topic of imaging prescription under the context where both options are considered standard of care, was used to select variables for inclusion in final multivariable model. All analyses were performed using Stata, and values with *p* < 0.05 were considered statistically significant.

## 3 RESULTS

### 3.1 Patient characteristics

The patient characteristics of the study sample are summarized in Table 1. Among the overall study sample, 79.1% were non‐Hispanic White (White), 2.1% were non‐Hispanic Black (Black), 8.5% were Asian/Pacific Islander (Asian), and 10.3% were Other. Due to the small number of non‐White patients in the sample, we collapsed the race category into two groups: White and non‐White. Approximately, 2.1% of patients were between 45 and 54, 20.9% of patients were between 55 and 64, 43.2% of patients were between 65 and 74, 29.1% of patients were between 75 and 84, and 4.7% of patients were 85 years and above. The mean age in the study was 71 years (SD 8.2). Insurance status was categorized Medicare/Medi‐Cal or Public Insurance (74.8%), Private Insurance (23.1%), and OOP Payer (2.1%).

**Table 1:** Patient characteristics

	Overall (*N* = 234)		Conventional (*N* = 165)		Molecular (*N* = 69)	
	*N*	%	*N*	%	*N*	%
Age						
45–54	5	2.1	2	1.2	3	4.4
55–64	49	20.9	31	18.8	18	26.1
65–74	101	43.2	76	46.0	25	36.2
75–84	68	29.1	47	28.5	21	30.4
>85	11	4.7	9	5.5	2	2.9
Race						
White or Caucasian	185	79.1	125	75.8	60	87.0
Asian	20	8.5	16	9.7	4	5.8
Black or African American	5	2.1	4	2.4	1	1.4
Other	24	10.3	20	12.1	4	5.8
Insurance payer						
Private insurance	54	23.1	30	18.2	45	65.2
Public insurance	175	74.8	130	78.8	24	34.8
Out of pocket payment	5	2.1	5	3.0	0	0
Gleason score						
Low (≤6)	30	12.8	21	12.7	9	13.0
Intermediate (7)	113	48.3	81	49.1	32	46.4
High (8–10)	91	38.9	63	38.2	28	40.6
Treatment prior to imaging						
Radical prostatectomy (RP)	75	32.1	42	25.5	33	47.8
Definitive radiation	63	26.9	53	32.1	10	14.5
RP and radiation	96	41.0	70	42.4	26	37.7
Received any further treatment after imaging						
No	88	37.6	64	38.9	24	34.8
Yes	146	62.4	101	69.2	45	65.2
Received ADT after imaging						
No	100	42.7	73	44.2	27	39.1
Yes	134	57.3	92	55.8	42	60.9
Received radiation therapy after imaging						
No	180	76.9	130	78.8	50	72.5
Yes	54	23.1	35	21.2	19	27.5
Receipt of ADT prior to scan						
No	124	53.0	81	49.0	43	62.3
Yes	110	47.0	84	51.0	26	37.7
PSA prior to scan						
<0.2	19	8.2	11	6.7	8	11.6
≥0.2–<0.4	34	14.5	9	5.5	25	36.2
≥0.4–1	34	14.5	25	15.1	9	13.1
>1	147	62.8	120	72.7	27	39.1
Charlson comorbidity score						
0–2	147	62.8	103	62.4	44	63.8
3–6	76	32.5	57	34.5	19	27.5
≥7	11	4.7	5	3.1	6	8.7
Receipt of previous molecular imaging						
No	232	99.1	163	98.8	69	100
Yes	2	0.9	2	1.2	0	0
Number of previous scans						
0–2	7	3.0	7	4.2	0	0
3–4	156	66.7	91	55.2	65	94.2
≥5	71	30.3	67	40.6	4	5.8
Total payments						
$0–$5000	78	33.3	63	38.2	15	21.7
$5000–$10,000	36	15.4	26	15.8	10	14.5
$10,000–$500,000	90	38.5	54	32.7	36	52.2
$500,000–$1 million	10	4.3	7	4.2	3	4.3
>$1 million	20	8.5	15	9.1	5	7.2

Among the study population, 12.8% had low, 48.3% had intermediate, and 38.9% had high Gleason Scores. Approximately, 62.8% of patients had a PSA >1 ng/dL, and the mean PSA was 7.3 ng/dL (SD 20.0). Approximately 32.1% of the study population had prior RP, 26.9% had prior definitive radiation and 41.0% had prior RP and radiation. Half of the patients in the study sample had prior ADT. In the study sample, 0.9% had prior molecular imaging and the majority (97.0%) had more than two prior scans. The distribution for healthcare cost was wide, with 33.3% of patients paying $0–$5000, with a mean cost of $291,625 (SD 798,824).

### 3.2 Multivariate regression models

In Table 2, the primary results of the multivariate regression models in this study are presented. Model 1 reports the odds ratios for receipt of molecular imaging. Model 1 indicates that a one‐unit increase in PSA level is associated with the 21% lower likelihood of receiving molecular imaging (OR = 0.79) and higher odds of receiving conventional imaging. On the other hand, a one‐unit reduction in PSA level is associated with 1.3 times likelihood of receiving molecular imaging (*p* < 0.01). We observed that the scan types were not driven by insurance type. An additional McNemar's test was conducted to examine the marginal frequencies of two variables, insurance type and scan type. We could not reject the null hypothesis that the scan types were utilized for different insurance types at the same rate (*p* < 0.51). All other control variables were found to be not statistically significant.

**Table 2:** Multivariate regression models. Model 1 presents odds ratios for receipt of molecular imaging. Model 2 presents coefficients, which capture the association between the determinants and healthcare cost

	(1)	(2)
Variables	Odds ratio for molecular imaging^a^	Coefficient for total payments^b^
Scan type (*baseline* =* conventional*)		−56,167.622
Molecular imaging		(112,979.027)
Insurance payer (*baseline *= *Public insurance*)	1.745	533,206.507***
Private insurance	(0.749–4.069)	(139,200.829)
Race	0.521	195,684.737
	(0.215–1.262)	(123,881.112)
Age	1.003	−5774.960
	(0.955–1.052)	(7582.072)
Charlson score	1.131	−12,172.134
	(0.941–1.358)	(28,215.650)
Gleason score (*baseline* = *low*)		
Intermediate	0.679	11,090.459
	(0.250–1.845)	(159,805.606)
High	1.060	−101,492.518
	(0.382–2.946)	(163,951.594)
Prior PSA level	0.785***	6254.377**
	(0.689–0.894)	(2637.899)
Received prior molecular imaging	1.021	−2754.476
	(0.212–4.910)	(237,508.394)
Received radical prostatectomy	1.636	75,853.560
	(0.252–10.628)	(285,328.621)
Received definitive radiation	0.898	−144,146.933
	(0.144–5.610)	(279,469.155)
Received radical prostatectomy and prostate radiotherapy		
Constant	0.402	548,825.982
	(0.006–25.683)	(665,824.998)
Observations	234	234
*R*‐squared		0.159

^a^ 95% confidence interval for odds ratio in parentheses.

^b^ Standard error for coefficient in parentheses.

*** *p* < 0.01,

** *p* < 0.05,

* *p* < 0.1.

Model 2 in Table 2 presents the association between the determinants and the secondary outcome, where we evaluated the association between scan type and healthcare cost, where healthcare cost was log transformed for analysis to mitigate skewness in the variable. The analysis showed that scan type did not have a statistically significant relationship with healthcare cost. However, primary insurance type had a statistically significant and substantively meaningful relationship with healthcare cost. On average, patients with private insurance have $533,207 higher healthcare cost (*p* < 0.01) as compared to patients with public insurance. One unit increase in PSA level was also associated with an additional $6254 in healthcare cost (*p* < 0.05) and an increase in total payments by 2.1%. All other control variables were not statistically significant.

An additional analysis was conducted to evaluate if the last treatment before imaging (RP, prostate radiotherapy, or RP followed by radiation therapy) impacts the type of imaging patients received. In Table 3, Model 1 evaluated the association between last treatment prior to imaging and reported the odds ratios. The findings indicate that patients who received prostate radiotherapy only are 61% less likely to receive molecular imaging than those who received RP only (*p* < 0.10). We observed that patients who received both RP and prostate radiotherapy are 52% less likely to receive molecular imaging than those who received RP only (*p* < 0.10). However, these results did not meet the threshold for statistical significance as established in the methods of this study. No other control variables were found to be statistically significant.

**Table 3:** Results from multivariate logistic regression presented in odds ratio for receipt of molecular imaging as compared to conventional imaging, depending on initial definitive treatment

	(1)
Variables	Odds ratio for molecular imaging
Prior treatment (*baseline* = *Radical prostatectomy)*	
Definitive radiation	0.388*
	(0.150–1.004)
Radical prostatectomy and prostate radiotherapy	0.479*
	(0.227–1.009)
Insurance payer (*baseline *= *Public insurance*)	1.910
Private insurance	(0.807–4.522)
Race	0.579
	(0.238–1.404)
Age	1.012
	(0.963–1.063)
Charlson score	1.158
	(0.961–1.394)
Gleason score (*baseline* = *low*)	
Intermediate	0.843
	(0.300–2.367)
High	1.201
	(0.424–3.405)
Prior PSA level	0.785***
	(0.689–0.893)
Received prior molecular imaging	0.760
	(0.158–3.654)
Constant	0.385
	(0.009–16.243)
Observations	234
*R*‐squared	

95% confidence interval in parentheses.

*** *p* < 0.01,

** *p* < 0.05,

* *p* < 0.1.

## 4 DISCUSSION

This study takes the first step in understanding the current patterns of imaging utilization in a disease state where the standard of care includes both conventional and molecular imaging. We observed that low PSA level is significantly associated with receipt of molecular imaging over conventional imaging. Moreover, the subsequent healthcare costs for the patients evaluated were driven by insurance type and not by imaging type. Additionally, we observed in our dataset that the scan types received by patients were not driven by the patient's insurance type. While these observations will need to be explored with a larger, more diverse dataset, this novel study suggests that clinical, rather than social factors, inform the selection of imaging used in the context of BCR PCa.

Precision medicine in PCa treatment entails increased utilization of technologies such as genomic testing and molecular imaging in order to develop an individualized treatment plan for patients. In PCa, most clinical trials characterize stage of disease using conventional imaging and therefore conclusions on management are extrapolated based on disease stage observed in molecular imaging. Given the concern for a stage shift and increased utilization of intensified therapies for patients receiving molecular imaging, we sought to evaluate the subsequent healthcare costs for patients receiving conventional versus molecular imaging.

In this study, we did not observe a significant relationship between healthcare cost and imaging type. Rather, the healthcare cost was driven by primary insurance type. The observation that patients with private insurance have higher healthcare costs is consistent with prior research. In cancer treatment, Thorpe and colleagues reported that uninsured patients incurred 55% of the healthcare spending of privately insured patients.^10^ In general, reimbursement from public insurances tend to be lower than private insurances.^11^ Furthermore, these observations may be explained in part by patients with higher cost‐sharing or OOP costs seeking fewer provider visits, procedures, and medical attention in order to reduce cost. However, these cost differences are even observed in the context of emergency care. Jackson reported that among patients receiving care in the emergency department, privately insured patients carried higher discharge costs than uninsured patients.^12^

In our dataset, we also observed that there were also a very small number (*n* = 5) of patients who paid any OOP amount, therefore we were unable to determine total patient financial burden. Future research will need to more rigorously examine subsequent costs associated with receipt of molecular imaging.

Additionally, we observed that a reduction in PSA level was associated with a significantly higher likelihood of receiving molecular imaging. This result was not surprising as the median PSA level for those who received conventional imaging was 3.55 ng/dL, which was significantly higher than 0.41 ng/dL, which was the median PSA level for those who received molecular imaging in our study sample. Prior research examining the role of molecular imaging in this disease state described similarly low PSA levels at time of imaging. For example, the LOCATE trial which reported the impact of Fluciclovine PET on management of BCR PCa had a median PSA of 1 ng/dL among study patients.^13^ Hope and colleagues reported on the impact of PSMA PET imaging in the management of BCR PCa and reported an average PSA of 5.9 ng/dL, however approximately 33% of their patient sample had a PSA <2 ng/dL at time of imaging.^14^ Given that higher PSA levels are associated with detection of tumor on conventional scans,^15^, ^16^, ^17^, ^18^ using molecular imaging when biomarkers are low to detect low volume recurrent disease is consistent with current evidence‐based guidance on management of this disease state.^19^

This analysis examined different determinants that may influence provider utilization of imaging, paying special attention to factors that contribute to disparity. While we found that race was not a statistically significant determinant to imaging type, we recognize the limitations of a single site study, where the majority of patients were white. Therefore, it is necessary to conduct a similar analysis with a more diverse population. To date there are limited data characterizing disparities in receipt of molecular imaging using a real‐world dataset. Galgano and colleagues described the patient demographics of patients referred for Fluciclovine PET imaging at their institution and observed that that African American men were under‐referred for molecular imaging.^20^ Future studies will need to explore if racial/ethnic disparities exist using a larger more diverse dataset. Given that molecular imaging is increasingly becoming the standard of care in this disease state, attention to potential disparities in utilization patterns is critical.

This study takes the first step in evaluating determinants of imaging prescription in the context where both imaging options are considered the standard of care. Relying on retrospective data this study focuses on examining the associations between potential determinants and imaging type but does not evaluate the causal framework. Another limitation of this analysis is that recent data suggest that Fluciclovine PET imaging may be inferior to detecting low volume recurrent disease compared to PSMA imaging in the BCR PCa setting,^21^ however given that both imaging modalities are routinely ordered at time of recurrence this analysis combined all molecular imaging available at the study site. Moreover, given that the PSMA imaging is considered investigational, there may be selection bias in utilization of this imaging modality at the study site, but given that PSMA imaging was made available at the institution, the information allowed us to take the first step at evaluating the pattern of utilization as PSMA is introduced as a prescription option. Lastly, the healthcare cost data only include cost in outpatient settings as inpatient data are not itemized and do not allow for evaluation of prostate cancer specific costs. Nevertheless, the outpatient data provide valuable information on the varying cost incurred by patients as part of prostate cancer care.

Despite these limitations, this study has numerous strengths. To our knowledge, this is the first attempt to describe determinants of receipt of molecular imaging in BCR PCa. We believe that this study adds to the emerging literature on the role of molecular imaging in prostate cancer management, with attention to the implications on costs of care as we were able to identify potential drivers of healthcare costs in our study sample.

## 5 CONCLUSIONS

This study identified low PSA level is significantly associated with subsequent receipt of molecular imaging in prostate cancer with biochemical recurrence. We also observed that high healthcare costs were driven by insurance type in this patient population. In the future, attention to the role of social factors such as race/ethnicity in receipt of molecular imaging will need to be examined.

## CONFLICTS OF INTEREST

All authors have no disclosures or conflicts of interest.

## AUTHOR CONTRIBUTIONS

Study concept and design: Borno, Desai, Koshkin, Lin, Zhang; Acquisition of data: Borno, Odisho, Legaspi, Bell, Lin, Zhang; Analysis and interpretation of data: Borno, Lin, Werner, Hope; Drafting of the manuscript: Borno, Lin; Critical revision of manuscript: Borno, Lin, Odisho, Desai, Koshkin, Bucknor, Legaspi, Bell, Werner, Zhang, Hope; Statistical analysis: Lin, Werner; Other: None.

## Data Availability

Data are available upon request.
